# Testing skin swabbing for DNA sampling in dendrobatid frogs

**DOI:** 10.1163/15685381-17000206

**Published:** 2018-04-24

**Authors:** Eva Ringler

**Affiliations:** 1 Department of Integrative Biology and Physiology, University of California Los Angeles, 621 Charles E. Young Drive South, Los Angeles, CA 90095-1606, USA; 2 Department of Integrative Zoology, University of Vienna, Althanstrasse 14, A-1090 Vienna, Austria; 3 Messerli Research Institute, University of Veterinary Medicine Vienna, Medical University of Vienna, University of Vienna, Veterinaerplatz 1, A-1210 Vienna, Austria

**Keywords:** *Allobates femoralis*, amplification success, Dendrobatidae, DNA yield, non-invasive sampling, skin swab

## Abstract

Skin swabbing, a minimally invasive DNA sampling method recently proposed for adult amphibians, was tested on the dendrobatid frog *Allobates femoralis*. I compared DNA yield from skin swabs and toe clips by evaluating obtained DNA concentrations and purity of extracts, as well as amplification success using eleven polymorphic microsatellite loci. I also tested whether storing skin swabs for two months at −20°C affected the properties of the extract or microsatellite analysis. Results show that skin swabs of adult *A. femoralis* suffered from high contamination and yielded significantly lower DNA quality and quantity, resulting in insufficient genotyping success, than DNA obtained from toe clips. The relatively dry skin in dendrobatid frogs may have impeded the collection of sufficient viable cells, and the presence of skin alkaloids and microbiota in the frog mucus may lead to high contamination load of skin swabs.

Molecular genetic analyses have become an essential and very powerful tool for studying the behaviour, ecology and evolution of animal populations. Cells containing DNA of the individuals of interest can be obtained in several ways. “Invasive sampling” is defined as the collection of blood or the removal of parts of an animal’s body tissue. Genetic material can also be obtained less invasively from hair roots, feathers, faeces, or via various swabbing techniques (Waits and Paetkau, 2005).

In mammals and birds, sampling hairs and feathers, respectively, is relatively easy, and these samples will usually provide sufficient cells for obtaining useful amounts of DNA (Goossens et al., 1998; Handel et al., 2006). Amphibians lack such easily accessible tissue, thus sampling amphibian DNA usually involves invasive approaches such as collecting blood or removing toes or pieces from the tail. Traditionally, toe clipping was not performed to collect genetic material from amphibians but rather to label individuals for mark-recapture studies (Donnelly et al., 1994). After some studies demonstrated that removing multiple toes decreased individual survival rates (Davis and Ovaska, 2001; Parris and McCarthy, 2001; McCarthy and Parris, 2004), clipping toes as a marking procedure for amphibians has raised increasing ethical and conservation concerns (Funk et al., 2005). Although the removal of only one toe or toe tip, as would be required for genetic studies, did not show detrimental effects on many species (McCarthy and Parris, 2004; Grafe et al., 2011; Ursprung et al., 2011a), researchers are still looking for less invasive ways to collect genetic material in amphibians. Such minimally invasive approaches have become a major focus of research, particularly since amphibians have been recognized as the most threatened vertebrate group with dramatically declining population sizes (Stuart et al., 2004; Beebee and Griffiths, 2005).

For amphibians, buccal swabbing (Pidancier et al., 2003; Broquet et al., 2007; Gallardo et al., 2012), skin swabbing (Prunier et al., 2012; Müller et al., 2013; Pichlmüller et al., 2013), as well as cloacal swabbing (Müller et al., 2013; Pichlmüller et al., 2013) have been proposed as potentially suitable sampling procedures, but have yielded inconsistent results across species and techniques. For example, buccal swabbing was successful in several species of frogs, but might not be easily applicable to small amphibian species, as opening the jaw and collecting buccal cells with cotton swabs may pose a severe injury risk. Buccal sampling frequently causes bleeding in the mouth regions of amphibians (Pidancier et al., 2003; Poschadel and Möller, 2004; own observation), and the presence or absence of blood in the sample might have caused the high variation in DNA quantities that have been reported in these studies. Also cloacal swabbing might not constitute a suitable sampling procedure for small amphibians as a cotton swab has to be inserted into the cloacal region. Moreover, a recent study in *Salamandra salamandra* reported very low success in obtaining good quality DNA from cloacal samples (Pichlmüller et al., 2013).

Skin swabbing, in turn, might be a more reasonable approach for small amphibians. It also requires less handling time, and thereby probably causes less stress and potential injuries to the animal compared to buccal swabbing. Skin swabbing for DNA sampling so far has been applied to a range of amphibians (newts and tree frogs, Prunier et al., 2012; water frogs, Müller et al., 2013; salamanders, Pichlmüller et al., 2013). Moreover, it has proven successful for other purposes such as detection of chytridiomycosis (Kriger et al., 2006) or the sampling of microbiota (Sabino-Pinto et al., 2016).

However, these species are rather big and/or feature very moist skin that may facilitate the collection of skin cells (cf. Prunier et al., 2012). To date, there is no information on how skin swabbing works in small terrestrial species such as poison frogs. In this study, I aimed to test previously published protocols for DNA sampling from skin swabs when applying them to a species with different skin properties. To this end, I collected skin swabs as well as toe clips of the dendrobatid frog *Allobates femoralis* to (i) compare the quantity and quality of extracted DNA, (ii) investigate the effect of sample storage on DNA yield, and (iii) quantify the respective success and reliability of microsatellite amplification.

*Allobates femoralis* is a small diurnal leaf litter frog that is distributed across the Amazon basin and Guiana shield (Amezquita et al., 2009; Fouquet et al., 2012). Males are highly territorial throughout the prolonged reproductive period that coincides with the local rainy season (Gottsberger and Gruber, 2004). Terrestrial clutches are deposited inside male territories, and after about 3 weeks the fathers transport the hatched tadpoles to water pools (Ringler et al., 2013a). Apart from the tadpole deposition no other activities in *A. femoralis* take place in or even near to water. Previous studies have used toe clips of adults as well as tail-fin clips of tadpoles to determine parent-offspring relationships, calculate pairwise genetic relatedness between individuals, and genetically track individuals across life history stages (Ursprung et al., 2011b; Ringler et al., 2012; Ringler et al., 2015a; Ringler et al., 2015b). In the present study, I aimed to assess the suitability of skin swabs and already published protocols for collecting genetic material in this species. Given their terrestrial lifestyle and the high territoriality of the males, I expected the risk of cross-contamination between individuals to be rather low.

On 7 March 2017, a total number of 10 individuals (5 males, 5 females) were randomly selected from the laboratory *A. femoralis* population housed at the animal care facilities at the University of Vienna. DNA was collected using sterile collection swabs with a cotton tip and wooden handle (e.g. type “300200”, DELTALAB, Rubí, Spain). The dorsal, lateral and ventral sides of all individuals were swabbed twice to collect skin cells. Two such skin swabs were collected per individual. One sample was immediately used for a genomic DNA extraction (“fresh”), the other one was stored dry at –20°C for 2 months before DNA extraction (“frozen”). The two swabs were assigned to one of the two storage groups in an alternating order. Additionally, toe clips were taken from all individuals, following Ursprung et al. (2011a). Toe clips were stored in 96% ethanol and kept at room temperature until extraction. DNA was extracted from all samples by using a phenol-chloroform procedure after proteinase K digestion (Sambrook et al., 1989). The DNA pellet was resolved over night at room temperature by adding 50 *μ*l milliQ H_2_O. The yield and purity of DNA was quantified using a Nanodrop 2000c spectrophotometer (Thermo Fisher Scientific). For each sample three measurements were taken and mean values were calculated for the obtained measures of DNA quantity (ng/*μ*l), as well as purity (260/280 and 260/230 ratios). The ratio of absorbance at 260 nm and 280 nm typically indicates the purity of a given DNA sample, with ratios of ~1.8 commonly being considered as “pure”. Considerably lower values may indicate the presence of contaminants such as proteins and phenol. Also the ratio 260/230 provides a suitable measure of nucleic acid purity, with expected values commonly in the range of 2.0-2.2. I then investigated the amplification success of each sample at 11 microsatellite loci (supplementary table S1, following protocols of Jehle et al., 2008; Ursprung et al., 2011b; and Ringler et al., 2013b; for details see online supplement) and compared allelic dropout rate (ADO), false allele rate (FA), and the resulting genotyping error rate (GER = ADO + FA) across samples. Data were analysed using SPSS 24.0 for Windows (IBM Corp. Release 2016). Normality of parameters was tested with the Kolmogorov-Smirnov test (supplementary table S2). Where parameters deviated significantly from a normal distribution, non-parametric measures such as the median and corresponding interquartile ranges (iqr) are given. Kruskal-Wallis tests were conducted to test for differences between sample groups, and Mann-Whitney *U* tests were applied for post-hoc pairwise comparisons. Resulting *p*-values were adjusted according to Bonferroni correction to account for multiple testing. Alpha for rejection of null hypotheses was set a priori at *p* < 0.05.

DNA concentrations, as given by the nanodrop measures, ranged from 51.9-167.8 ng/*μ*l (mean ± SD = 90.7 ± 35.3 ng/*μ*l) in toe clips, from 77.3-244.9 ng/*μ*l (mean ± SD = 128.9 ± 46.8 ng/*μ*l) in fresh skin swabs, and from 20.3-114.5 ng/*μ*l (mean ± SD = 58.7 ± 26.7 ng/*μ*l) in frozen swabs (fig. 1a). DNA concentration measures were significantly different across samples (Kruskal-Wallis test: *H*_2_ = 14.325, *p* = 0.001), with significant differences only between fresh and frozen swabs (Mann-Whitney *U* test, *U* = 14.9, adjusted *p*-value < 0.001, supplementary table S3). Also the 260/280 ratios (“toes”: mean ± SD = 1.97 ± 0.07; “fresh”: mean ± SD = 1.80 ± 0.18; “frozen”: mean ± SD = 1.86 ± 0.27) were within the range of “pure” DNA, and did only marginally differ across samples (Kruskal-Wallis test: *H*_2_ = 5.784, *p* = 0.055, supplementary table S3). However, when looking at the 260/230 ratios and the spectral patterns of the samples (fig. 1b and c and supplementary fig. S1), both skin-swab groups suffered from high contamination load. While the 260/230 values for the toe clips were well within the range of “pure” DNA (median = 1.99, iqr = 1.88-2.15), the values for fresh (median = 0.14, iqr = 0.13-0.15) and frozen swabs (median = 0.13, iqr = 0.11-0.18) were considerably lower and significantly different from the values of the toe clips (Kruskal-Wallis test: *H*_2_ = 19.467, *p* < 0.001; pairwise comparisons: toe versus fresh: *U* = 14.5, adjusted *p*-value = 0.001; toe versus frozen: *U* = 15.5, adjusted *p*-value < 0.001, supplementary table S3). As only skin swabs featured these low purity values, but all samples were extracted by the same protocol, I can rule out that contamination caused by the extraction method (e.g. phenol) could have led to the observed results. The differences across sampling groups were most evident when looking at spectral patterns (table S1). While there was a pronounced peak at 260 nm in the toe samples, such a peak was absent in both skin swab samples, indicating high amounts of contaminants from either proteins, alkaloids and/or degraded DNA from the microbiome or the sample individual. Visual inspection of migration patterns of extracted DNA on 3% agarose gels confirmed that swab samples only contained very low amounts of genomic DNA (i.e. bands hardly visible).

Amplification success, calculated as the proportion of PCR reactions that led to a readable genotype, was significantly different across samples (Kruskal-Wallis test: *H*_2_ = 21.272, *p* < 0.001). Values ranged between 81.8% and 100% (median = 90.9, iqr = 90.9-90.9) for the toe samples, and were significantly lower in the fresh (median = 9.09, iqr = 0-15.9; *U* = 17.35, adjusted *p*-value < 0.001) and frozen skin swabs (median = 22.72, iqr = 9.09-34.09; *U* = 12.65, adjusted *p*-value = 0.01, fig. 1d, supplementary table S3). Consequently, the GER was extremely high when comparing both skin swab groups to toe clips (fresh: mean ± SD = 99.55 ± 1.44%; frozen: mean ± SD = 88.18 ± 13.24%), which was mainly caused by the high allelic dropout rate in both fresh and frozen skin swabs (fresh: mean ± SD = 91.82 ± 7.96%; frozen: mean ± SD = 79.09 ± 16.06%). Also the match between the few shared amplified loci was low. In fresh swabs only one out of the 18 corresponding amplified alleles matched the allele in the toe clips, whereas 26 out of 46 corresponding amplified alleles matched in frozen swabs and toe clips (see supplementary table S4).

In order to test if purification procedures could have improved the quality of the DNA, five fresh and five frozen samples were randomly selected and processed using a genomic DNA purification kit (DNeasy Blood & Tissue Kit, QIAGEN, Germany), with the standard protocol followed excluding cell lysis. However, after this step DNA quantity and quality measurements could not detect any remaining DNA, and also no microsatellite loci could be amplified from these samples.

Overall, the yield of DNA extracted from skin swabs did not allow reliable genotyping of microsatellite loci in our study species. I speculate that the relatively dry skin of dendrobatid frogs may hinder the collection of viable skin cells, instead yielding dead, sloughed cells that contain degraded DNA. A similar finding was made by Prunier et al. (2012), who found that the ventral skin of *Hyla arborea* was too dry to allow for a successful collection of viable skin cells. In addition, the presence of microbiota on (de Assis et al., 2017) as well as alkaloids inside the skin (cf. Amézquita et al., 2017) may further add perturbing contaminants to the skin swab samples. Although both microbiomes as well as skin defence compounds can become altered in captivity (Sabino-Pinto et al., 2016), I do not think that such alteration could have biased our results, since typically lower diversity is expected in captivity compared to the wild. That freezing the samples slightly enhanced amplification success compared to room temperature storage was also reported by Pidancier et al. (2003) when using buccal swabs. Although previous studies demonstrated that the amphibian skin can provide a valuable alternative source of DNA, particularly for sampling endangered species, still their application must be treated with caution. In general, samples yielded low DNA quantity, which negatively affected genotyping success and allele matching (Prunier et al., 2012; Müller et al., 2013). Sampling success may also be affected by observer experience (Pichlmüller et al., 2013). Moreover, skin swabs were found to suffer from strong contamination through other individuals (Müller et al., 2013), which considerably decreases their suitability for individual-based studies or next-generation sequencing approaches, which are particularly susceptible to such contaminations.

Minimally invasive methods for collecting DNA from individuals have become a major focus of interest for molecular ecology and conservation genetics studies (Taberlet et al., 1999; Beja-Pereira et al., 2009). Despite the urgent need for such approaches, we need to carefully evaluate the suitability of protocols to be applied to further species, and the reliability of obtained data.

The findings of this study highlight the importance of pilot experiments for evaluating the reliability and general applicability of certain sampling approaches prior to starting any given study. For example, the eventual exclusion of single samples due to limited amplification success might be less of a problem for population genetic studies, but may considerably impact any individual-based approaches. Eventually, other skin swabbing techniques and/or DNA extraction and purification approaches (e.g. sampling with flocked swabs, sample storage in RNAse, Salt extraction protocols) may retrieve higher quality DNA from skin swabs in dendrobatid frogs, which will need to be evaluated in future studies.

## Supplementary Material

Supplement

## Figures and Tables

**Figure 1:**
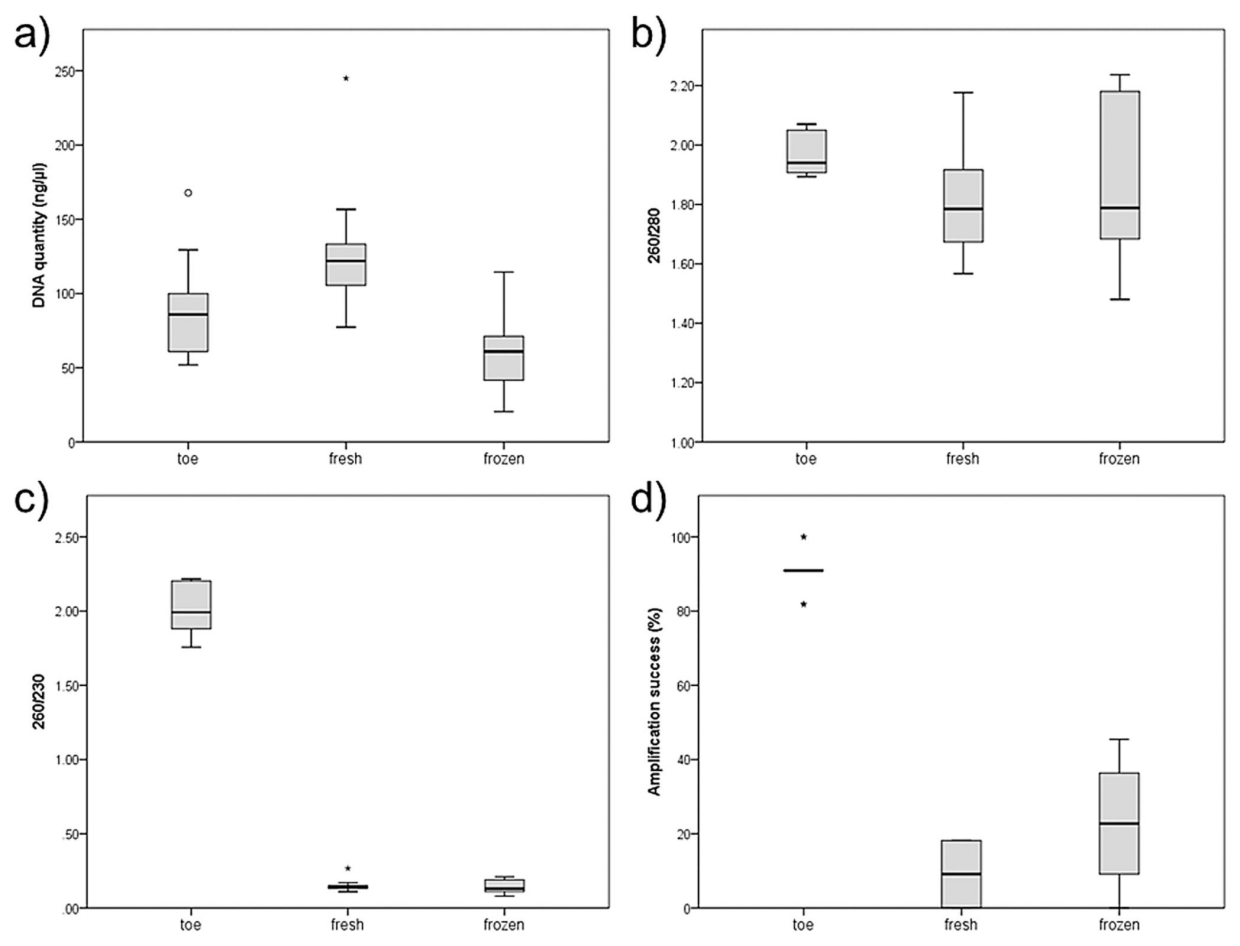
DNA quantity and purity as given by the Nanodrop measurements. Boxplots show (a) ranges of DNA quantity given in ng/*μ*l, (b) 260/280 ratios, (c) 260/230 ratios, and (d) amplification success of 11 microsatellite loci across toe clip samples (“toe”), immediately extracted skin swabs (“fresh”), and skin swabs that were stored for two months under −20°C before extraction (“frozen”).
