# Supplementary material for: Testing skin swabbing for DNA sampling in dendrobatid frogs
Source: Amphib Reptil. Author manuscript; Available in PMC 2019 Jul 19. (PMC6640035; doi:10.1163/15685381-17000206)
Supplement: Supplement [file EMS83544-supplement-Supplement.pdf]

Testing skin swabbing for DNA sampling in dendrobatid frogs.

Eva Ringler[1,2,3]

1 Department of Integrative Biology and Physiology, University of California Los Angeles, 621 Charles E. Young Drive South, Los Angeles, CA 90095-1606, USA
2 Department of Integrative Zoology, University of Vienna, Althanstrasse 14, A-1090 Vienna, Austria.
3 Messerli Research Institute, University of Veterinary Medicine Vienna, Medical University of Vienna, University of Vienna, Veterinaerplatz 1, A-1210 Vienna, Austria.

Supplementary material

Genotyping

PCR amplifications were performed using reaction volumes of 10 µl containing 10 ng of genomic DNA, 0.2 mM of each dNTP, 1 µM of each forward and reverse primer, 0.5 U of Taq DNA polymerase (Axon), 1 µl of 10×NH_4 reaction buffer (Axon), at a final concentration of 1.5 mM $MgCl_2$. Finally, we added 0.1 µl of Bovine Serum Albumin to each reaction. We used the following PCR program: 5 min at 95 °C, 35 cycles at 95 °C for 45 s, the primer specific annealing temperature (Table S1) for 45 s, 72 °C for 45 s, followed by a final extension step for 5 min at 72 °C. Differences in the sizes of the amplified products and in the fluorescent dyes of the primers allowed for pooling of multiple loci in the subsequent sequencing process. The pooled products were diluted with water 1:20, mixed with HiDiformamid and the internal size standard ROX500 (Applied Biosystems), and run on an ABI 3130xl Genetic Analyzer. Alleles were manually extracted with Peakscanner software (Applied Biosystems), and final allele sizes were determined using TANDEM v.1.08 (Matschiner & Salzburger, 2009). PCRs were repeated up to three times in case of low amplification success.

References

Matschiner M, Salzburger W (2009) TANDEM: integrating automated allele binning into genetics and genomics workflows. Bioinformatics **25**: 1982-1983.

Table S1. Details on the 11 microsatellite loci used in this study.

**Locus**	**Dye and primer sequence (5′–3′)**	**AT (°C)**	**GenBank accession no**
Afem03	F: (FAM)-ATGAACACAGAGCCGAGACC<br>R: TCAATATGTCAATGTATCTTTCAATC	58	EU414774
Afem04	F: HEX-GAGACGCCTGTTATAGATGGTG<br>R: TTAAAATGCACCGACACTGG	56	KF021569
Afem09	F: (TET)-TTCCCAACATCCCTACATCC<br>R: TCAATTCACAGCACCACTCC	57	EU414777
Afem12	F: (FAM)-GGCCTTTGTGACATGTGATG<br>R: CTCCAGGCCTCAAACAAGAG	58	EU414778
Afem13	F: (FAM)-TTTGTAGGTGGGAAAACTTGC<br>R: TCAGCTTCCAAGAATAAATACGG	58	EU414779
Afem15	F: (HEX)-GAATATTCAGTGTCCCGGAAG<br>R: GTGCCGCTCATTAAGCTCAT	56	EU414781
Afem16	F: (HEX)-ACAGGGTAGAGGCTTTGGTG<br>R: TCATTGAGGTCTTAGTTTTCCA	56	EU414782
Afem20	F: TET-TCCTAAGCCAGAGGAAGCTG<br>R: ACAATCACATGCACCGAGTC	56	KF021570
Afem22	F: NED-ACCGTGGAGTGGTTGATGAG<br>R: CCTGCCAAGGATTGATAAGC	52	KF021571
Afem24	F: NED-AAAGTAGGGTCGCAGCACTC<br>R: AGGTCAAGTCGGATGGTTTG	56	KF021573
Afem25	F: HEX-GTAATCCCCCAATCCTGGTC<br>R: GATCCCGGCATCGTTAAG	61	KF021575

Table S2. KS-test. Significant deviation from normal distribution is given in bold.

	n	$\boldsymbol{X^2}$	***P***
Quantity (ng/µl)	30	0.144	0.114
260/280	30	0.096	0.1
**260/230**	30	0.377	**< 0.001**
**Amplification success**	30	0.215	**0.001**

Table S3. Results of the Kruskal-Wallis tests and corresponding post-hoc Mann-Whitney U tests

		Mann-Whitney *U* test	Kruskal-Wallis test
Quantity	toes vs fresh	$U = -7.6$<br>adj. p-value = 0.161	$H_2 = 14.325$<br>$p = 0.001$
	toes vs frozen	$U = 7.3$<br>adj. p-value = 0.191	
	fresh vs frozen	$U = 14.9$<br>adj. p-value < 0.001	
260/280	-	-	$H_2 = 5.784$<br>$p = 0.055$
260/230	toes vs fresh	$U = 14.5$<br>adj. p-value = 0.001	$H_2 = 19.467$<br>$p < 0.001$
	toes vs frozen	$U = 15.5$<br>adj. p-value < 0.001	
	fresh vs frozen	$U = 1$<br>adj. p-value = 1	
AS	toes vs fresh	$U = 17.35$<br>adj. p-value < 0.001	$H_2 = 21.272$<br>$p < 0.001$
	toes vs frozen	$U = 12.65$<br>adj. p-value = 0.01	
	fresh vs frozen	$U = -4.7$<br>adj. p-value = 0.681	

Table S3. Microsatellite genotypes of all samples used in this study. ‘a’ and ‘b’ refer to the alleles of a given locus. ‘AS’ = amplification success, given in percent of amplified loci relative to the total number of loci ananlyzed; ‘ADO’ = allelic dropout rate, ‘FA’ = false allele rate, ‘GER’ = genotyping error rate (GER=ADO+FA) over all loci of a given sample.

Individual	Sex	Sample	12a	12b	9a	9b	3a	3b	15a	15b	13a	13b	16a	16b	20a	20b	24a	24b	4a	4b	25a	25b	22a	22b	AS	ADO	FA	GER
Femo079	F	toe	148	160	189	193	196	200	192	192	228	260	415	455	146	152	199	211	265	265	191	199	169	193	100.00	-	-	-
Femo079	F	fresh	0	0	0	0	0	0	0	0	0	0	0	0	0	0	0	0	0	0	0	0	181	189	9.09	90.91	9.09	100.00
Femo079	F	frozen	112	136	0	0	196	196	0	0	0	0	0	0	138	146	0	0	0	0	0	0	0	0	27.27	72.73	18.18	90.91
Femo170	M	toe	132	152	185	185	204	240	164	164	228	232	427	431	138	144	243	251	269	269	213	223	161	189	100.00	-	-	-
Femo170	M	fresh	0	0	0	0	0	0	0	0	0	0	0	0	0	0	0	0	0	0	0	0	181	189	9.09	90.91	4.55	95.45
Femo170	M	frozen	132	136	0	0	204	240	0	0	0	0	0	0	138	144	243	251	0	0	0	0	0	0	36.36	63.64	4.55	68.18
Femo081	F	toe	152	156	0	0	196	200	0	0	228	260	415	455	140	152	199	211	265	265	191	199	169	193	81.82	-	-	-
Femo081	F	fresh	112	136	0	0	0	0	0	0	0	0	0	0	0	0	0	0	0	0	0	0	181	189	18.18	81.82	18.18	100.00
Femo081	F	frozen	112	136	0	0	0	0	0	0	0	0	0	0	122	122	211	223	0	0	0	0	0	0	27.27	72.73	22.73	95.45
Femo175	F	toe	112	152	193	201	200	228	156	156	0	0	411	415	138	146	199	203	0	0	199	199	189	233	81.82	-	-	-
Femo175	F	fresh	0	0	0	0	0	0	0	0	0	0	0	0	0	0	0	0	0	0	0	0	0	0	0.00	100.00	na	100.00
Femo175	F	frozen	0	0	0	0	0	0	0	0	0	0	0	0	138	146	0	0	0	0	0	0	0	0	9.09	90.91	0.00	90.91
Femo116	F	toe	116	116	189	189	196	232	188	204	232	240	0	0	138	140	175	175	289	289	181	195	189	229	90.91	-	-	-
Femo116	F	fresh	0	0	0	0	0	0	0	0	0	0	0	0	0	0	0	0	0	0	0	0	0	0	0.00	100.00	na	100.00
Femo116	F	frozen	112	112	0	0	0	0	0	0	0	0	0	0	138	138	0	0	0	0	0	0	0	0	18.18	81.82	13.64	95.45
Femo073	F	toe	132	164	217	217	232	232	144	156	220	232	399	399	142	144	0	0	333	333	183	195	189	245	90.91	-	-	-
Femo073	F	fresh	0	0	0	0	0	0	0	0	0	0	0	0	0	0	0	0	0	0	0	0	193	193	9.09	90.91	9.09	100.00
Femo073	F	frozen	0	0	0	0	0	0	0	0	0	0	0	0	138	140	0	0	0	0	0	0	0	0	9.09	90.91	9.09	100.00
Femo097	M	toe	132	152	0	0	204	240	156	156	228	232	427	443	142	144	211	251	269	333	175	213	161	233	90.91	-	-	-
Femo097	M	fresh	0	0	0	0	0	0	0	0	0	0	0	0	0	0	0	0	0	0	0	0	0	0	0.00	100.00	na	100.00
Femo097	M	frozen	0	0	0	0	0	0	0	0	0	0	0	0	0	0	0	0	0	0	0	0	0	0	0.00	100.00	na	100.00
Femo172	M	toe	124	152	201	201	192	232	156	184	236	264	427	427	140	158	135	207	0	0	193	193	169	189	90.91	-	-	-
Femo172	M	fresh	0	0	0	0	0	0	0	0	0	0	0	0	0	0	0	0	0	0	0	0	0	0	0.00	100.00	na	100.00
Femo172	M	frozen	0	0	0	0	0	0	0	0	0	0	0	0	0	0	0	0	0	0	0	0	0	0	0.00	100.00	na	100.00
Femo128	M	toe	132	152	201	201	196	212	156	156	224	224	427	455	144	146	211	251	269	273	0	0	233	245	90.91	-	-	-
Femo128	M	fresh	0	0	0	0	0	0	0	0	0	0	0	0	138	138	175	223	0	0	0	0	0	0	18.18	81.82	18.18	100.00
Femo128	M	frozen	112	136	0	0	196	212	0	0	0	0	0	0	144	146	211	251	0	0	0	0	181	189	45.45	54.55	18.18	72.73
Femo044	M	toe	152	164	0	0	196	240	164	164	232	244	399	423	140	158	135	255	253	269	191	213	189	229	90.91	-	-	-
Femo044	M	fresh	0	0	0	0	0	0	0	0	0	0	0	0	138	138	223	223	0	0	0	0	0	0	18.18	81.82	18.18	100.00
Femo044	M	frozen	0	0	0	0	196	240	0	0	0	0	0	0	140	140	135	255	0	0	0	0	181	189	36.36	63.64	4.55	68.18

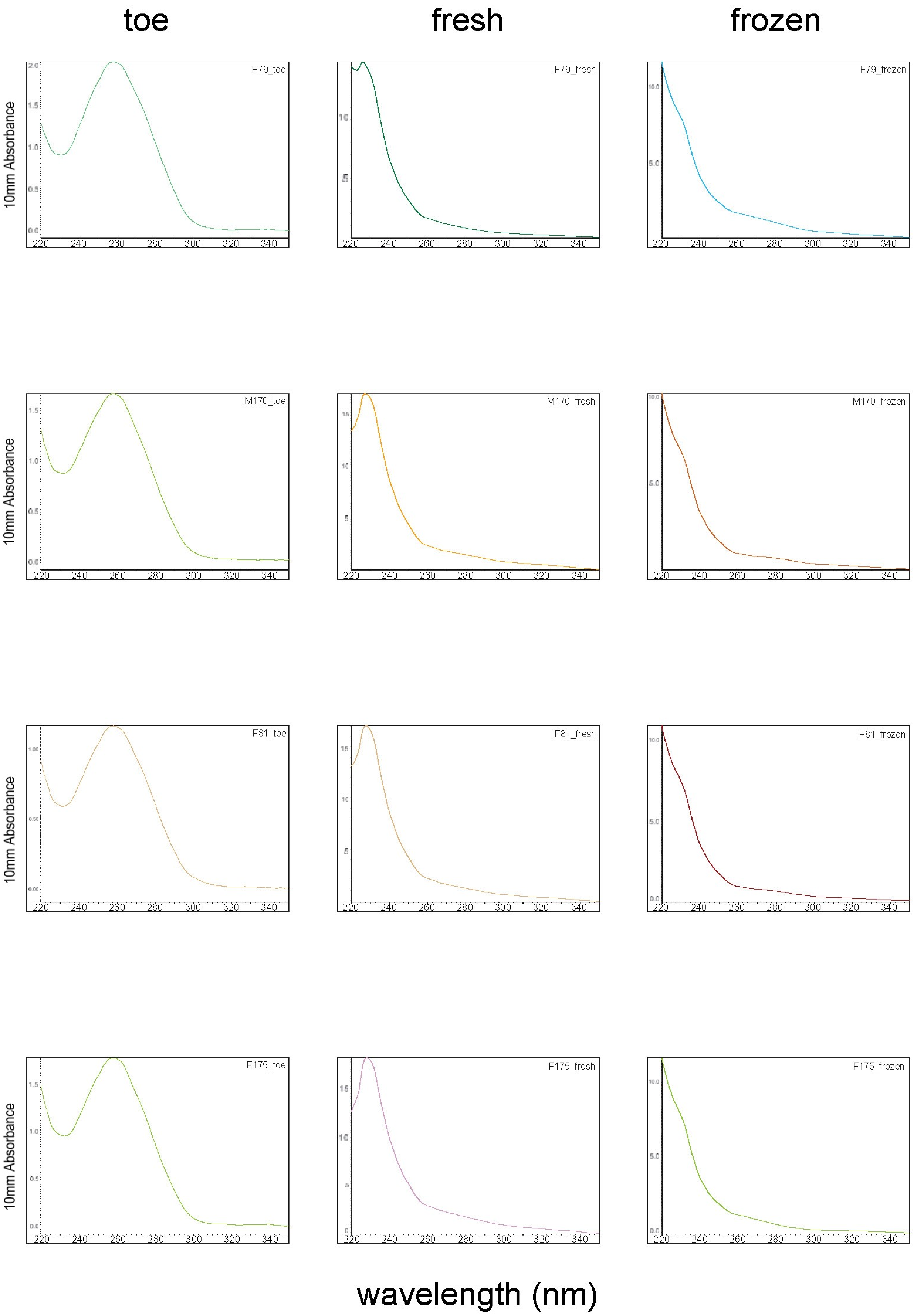
toe
fresh
frozen
10mm Absorbance
F79_toe
F79_fresh
F79_frozen
M170_toe
M170_fresh
M170_frozen
F81_toe
F81_fresh
F81_frozen
F175_toe
F175_fresh
F175_frozen
220
240
260
280
300
320
340
wavelength (nm)

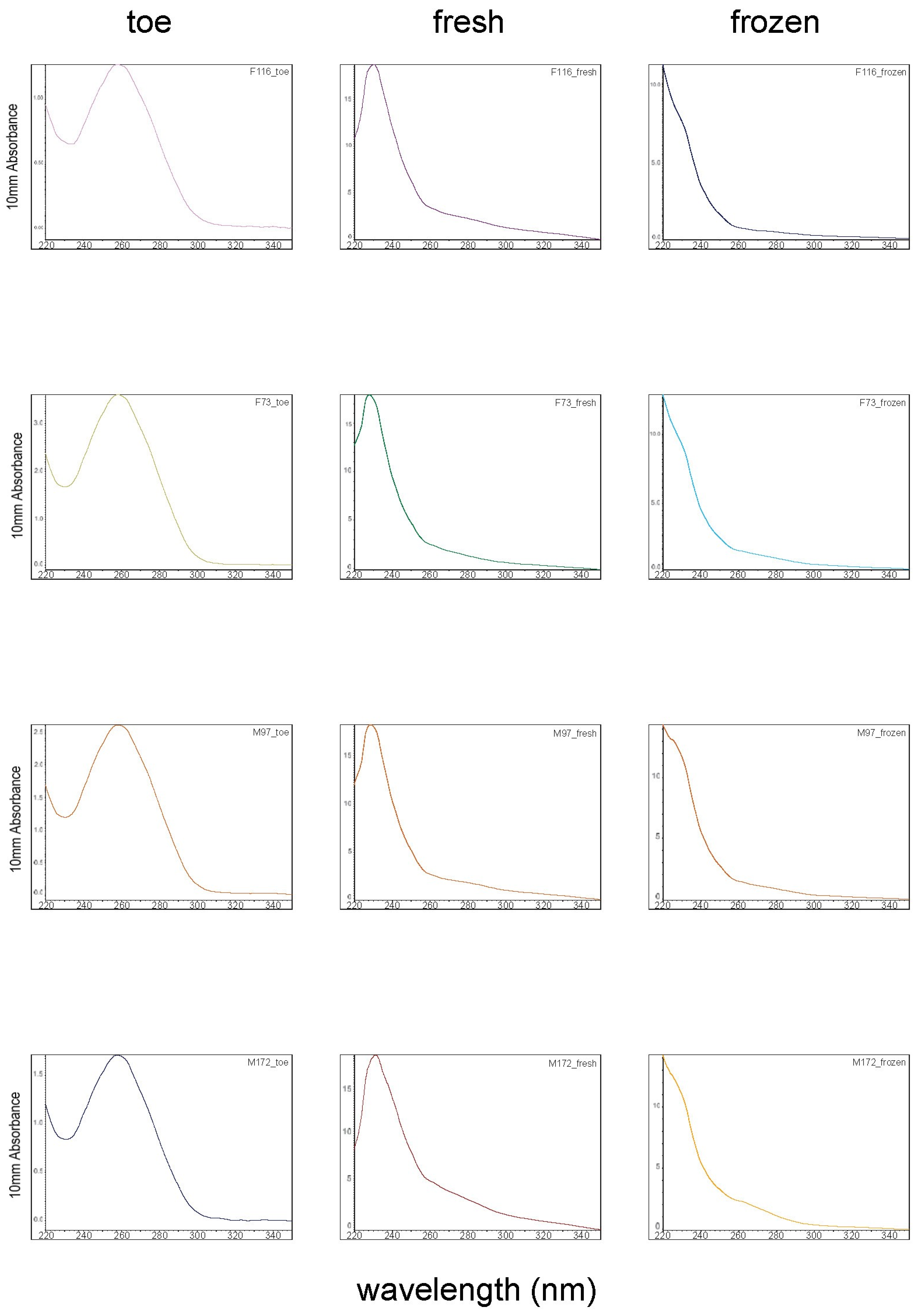

toe
fresh
frozen
10mm Absorbance
F116_toe
F116_fresh
F116_frozen
F73_toe
F73_fresh
F73_frozen
M97_toe
M97_fresh
M97_frozen
M172_toe
M172_fresh
M172_frozen
220 240 260 280 300 320 340
wavelength (nm)

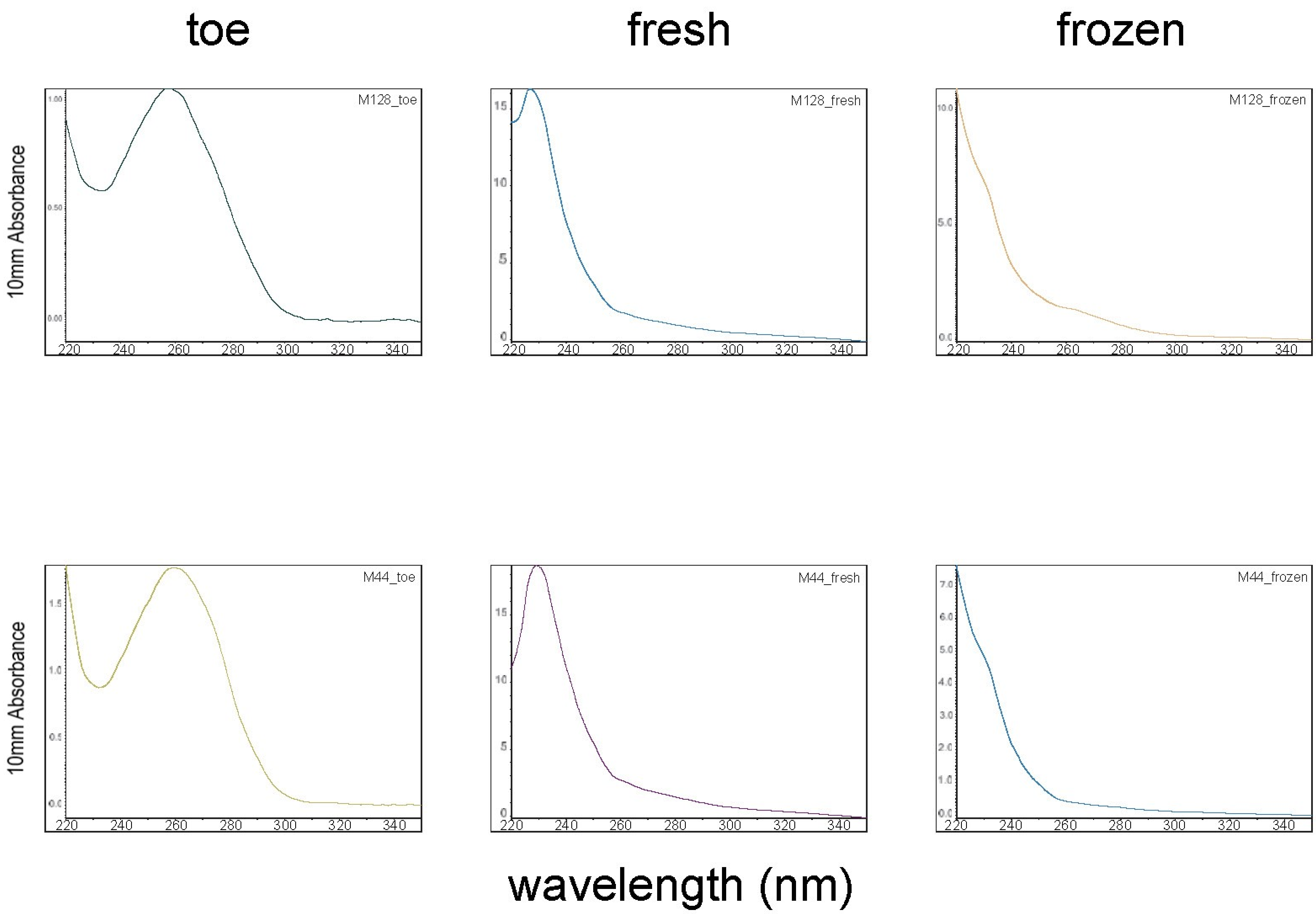

Figure S1. Spectral curves of all samples used in this study.
